# The impact of rainfall on the sea surface salinity: a mesocosm study

**DOI:** 10.1038/s41598-024-56915-4

**Published:** 2024-03-16

**Authors:** Lisa Gassen, Leonie Esters, Mariana Ribas-Ribas, Oliver Wurl

**Affiliations:** 1 Center for Marine Sensors (ZfMarS), Institute for Chemistry and Biology of the Marine Environment (ICBM), School of Mathematics and Science, Carl von Ossietzky Universität Oldenburg, Ammerländer Heerstraße 114-118, 26129 Oldenburg, Germany; 2 Institute for Geosciences, University of Bonn, Bonn, 53115 Germany; 3 Department of Earth Science, LUVAL, Uppsala University, Uppsala, 75236 Sweden

**Keywords:** Physical oceanography, Environmental sciences

## Abstract

Sea surface salinity may serve as a tracer for freshwater fluxes because it is linked to evaporation and precipitation that force the freshwater balance of the ocean’s surface. The relationship between freshwater fluxes and salinity anomalies in the upper few centimeters remains widely unknown. In a mechanistic approach, we investigated how these anomalies develop by conducting experiments with artificial rain over a large basin. We measured conductivity and temperature at different depths and rain characteristics (intensity, rain temperature, droplet sizes, and velocities). In the absence of turbulence, the rain causes a strong salinity change of up to 6.02 g kg$^{-1}$ in 0–4 cm depth. At the highest rain intensity of 56 mm h$^{-1}$, salinity changed thrice as fast as at an intensity of 18 mm h$^{-1}$. At the sea surface microlayer (first millimeter of the surface) the anomalies are always highest and reached a maximum of 14.18 g kg$^{-1}$ . With mechanical mixing, salinity changes were less pronounced (maximum SML salinity anomaly: 6.17 g kg$^{-1}$), and freshwater was mixed fast with the existing seawater body. In general, our study shows that freshwater remains in the upper few centimeters, and even with induced turbulence, are not mixed below 16 cm.

## Introduction

About 78% of the world’s precipitation and 86% of evaporation occur over the ocean^1,2^. This dominates one of the most crucial cycles on our planet: the global hydrological cycle^3–6^. The ocean surface, that is, the uppermost metre that includes the sea surface microlayer (SML), integrates changes in freshwater fluxes (i.e., evaporation minus precipitation), thus playing a key role in studying the oceanic hydrological cycle. Precipitation and evaporation determine temperature and salinity changes in the upper few layers of the mixed layer of the ocean. In particular, near-surface salinity is considered a tracer for detecting the processes of the oceanic hydrological cycle^7,8^. The SML is less than 1 mm thin^9^ and ubiquitously covers the global ocean as a surface film^10^. Because of its unique position, the SML is the place where the processes of freshwater fluxes instantaneously influence physical properties, such as sea surface salinity (SSS) and sea surface temperature (SST)^9,11,12^.

On one hand precipitation adds fresh water and lowers salinity at the sea surface. On the other hand, evaporation removes freshwater from surface waters and increases SSS. SST serves as a further tracer because precipitation usually has lower temperatures than the sea surface waters^13^ or air^14^. Furthermore, evaporation lowers the water temperature because of the latent heat flux^15^. SSS can serve as a proxy for freshwater fluxes integrating the variability of flux processes, and their spatially and temporal inhomogeneity, e.g., of turbulent latent fluxes^16^. However, the small-scale processes on the sea surface caused by precipitation and evaporation are very complex and can lead to unique physical structures and dynamics of the upper surface layers^17^. Depending on droplet size and velocity, the dynamics of raindrops can suppress capillary waves or prevent vertical mixing^18,19^. Small droplets have a low impact velocity and cannot break the surface, so they accumulate on top of the surface^20^. Larger droplets have a higher vertical velocity and can create vortex rings that penetrate the surface^21,22^. As the size of the droplets increases, the terminal velocity and momentum increase^23^. Not only do the properties of precipitation play a role, but the wind speed also influences how fast freshwater is mixed with the existing seawater body. At low wind speeds, freshwater lenses can form and persist for many days on the sea surface^24–26^ and lenses can accumulate freshwater over time because of multiple rainfall events and can reach the size of the rain front^27,28^.

Precipitation exhibits enormous spatial and temporal variability, making it difficult to obtain in situ data on temperature and salinity anomalies as a function of droplet properties. For this reason, the effects of rainfall intensity and droplet properties are not yet fully understood. We applied a nozzle-based rainfall simulation system to conduct rainfall experiments in a large seawater tank. Harrison and Veron^29^ conducted mesocosm studies with artificially generated rain in a wind wave flume to investigate the influence of rain on near-surface currents. In addition, mesocosm studies with artificial rain have been applied to investigate air–water gas exchange^18,30^. Our experiments do not aim to simulate natural rain but to obtain a mechanistic understanding of the distribution of rain-induced freshwater along the surface with a variation of rain intensities and raindrop properties. Our objective is to answer the following research questions: (i) How does freshwater from rainfall distribute vertically across the SML and near-surface layer in turbulence-free or/and turbulence-mixed water bodies? (ii) How do droplet sizes and velocities or/and rainfall intensity determine penetration depth of freshwater? (iii) How do properties of raindrops control mixing processes on the surface?

Our results focus on the role of SSS, but also discuss temperature as an important factor for density and buoyancy fluxes. Our results contribute to a better mechanistic understanding of the effects of precipitation on sea surfaces and show which depths are most affected by certain precipitation intensities or droplet properties. Such understanding is required to assess SSS as a key tracer for freshwater fluxes and to interpret in situ measurements in the future.

## Results

### Droplet characteristics

**Figure 1:**
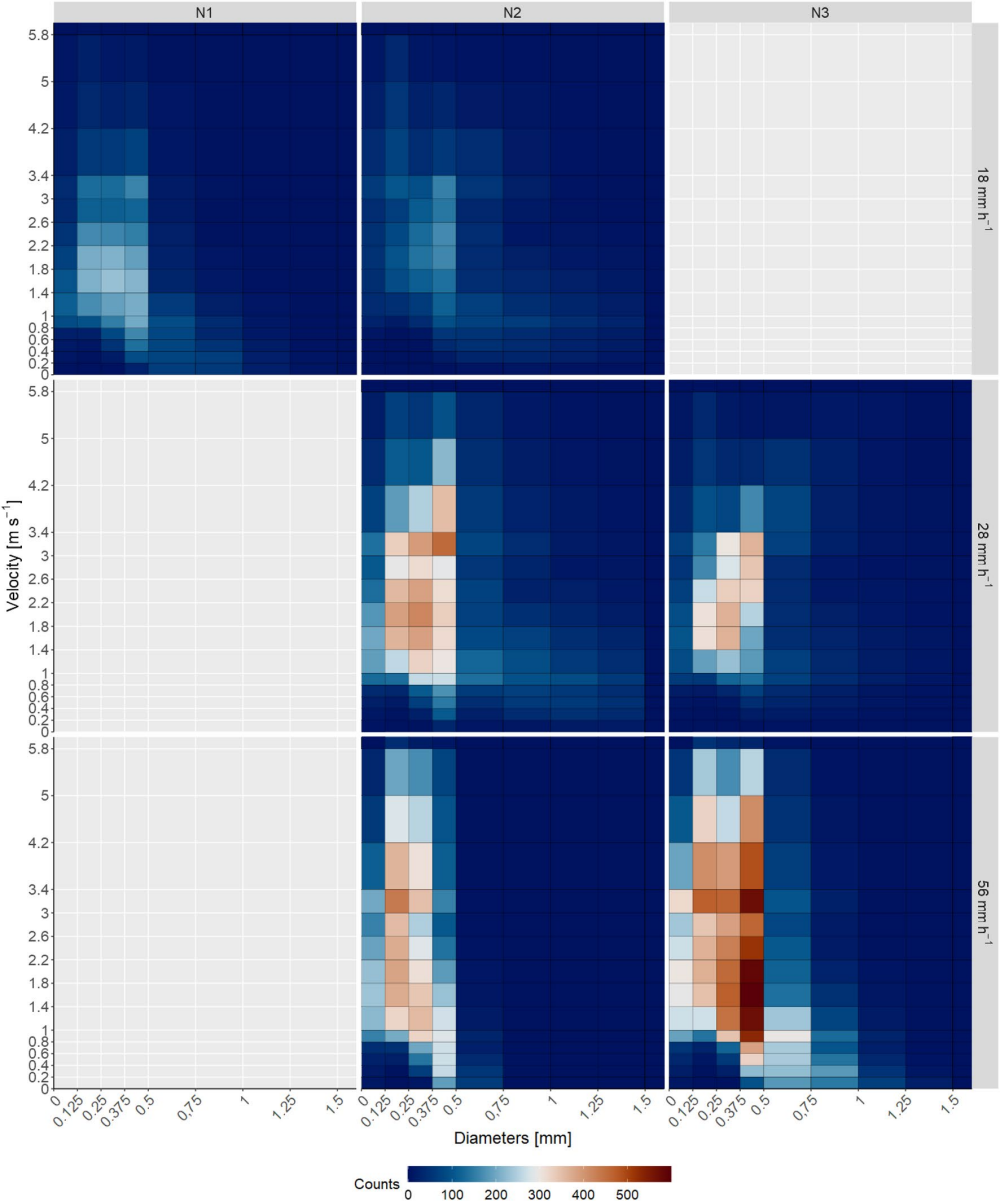
Distribution of droplet sizes and velocities of the three nozzle types N1, N2, and N3 and rain intensities 18 mm h^−1^, 28 mm h^−1^, and 56 mm h^−1^. The colours show the mean number of droplets of the size and velocity categories of 1 min.

Figure 1 shows the distribution of droplets of the three different nozzle types (N1, N2, and N3) and rainfall intensities 18, 28, and 56 mm h$^{-1}$, used in the rainfall simulation experiments. The droplets were categorized into 22 sizes and 20 velocities. In order to summarize the droplet velocity and size in a single parameter describing the impact of the droplets on the sea surface, the mean kinetic energy (KE) was calculated from the droplet numbers of different size and fall velocity categories (see “Methods” section). The results of KE for each nozzle type and rainfall intensity are shown in Table 1.

At a rainfall intensity of 18 mm h$^{-1}$, the N2 nozzle produced more droplets at higher velocities compared with the N1 nozzle, for example, more droplets with velocities greater than 4.2 m s$^{-1}$. In addition, the number of droplets with sizes greater than 1 mm was more pronounced with the N2 nozzle. At 18 mm h$^{-1}$, the N1 nozzle produced smaller droplets with diameters of 0–0.375 mm at low velocities, that is, between 0 and 1 m s$^{-1}$ (see Fig. 1). The high number of small droplets with lower velocities resulted in a lower mean KE over the entire experimental area for N1, with 11.38 J m$^{-2}$ h$^{-1}$ compared with N2, with a mean KE of 34.72 J m$^{-2}$ h$^{-1}$. The KE of the measurements directly below the nozzle, which was where the temperature and salinity measurements were made, was more than four times higher for the droplets produced with N2 (Table 1).

The number of larger droplets produced by N2 was higher than for N1 (see Fig. S1). The comparison between N2 and N3 at a precipitation intensity of 28 mm h$^{-1}$ shows that the N2 nozzle produced a higher number of droplets with larger diameters and velocities. The mean KE was also higher at 93.36 J m$^{-2}$ h$^{-1}$ compared with 77.62 J m$^{-2}$ h$^{-1}$. At a rainfall intensity of 56 mm h$^{-1}$, the N3 nozzle had higher counts of droplets with a larger diameter (> 0.5 mm) and droplets with higher velocities, compared to N2, here considering the mean counts of droplets for the entire area. The mean KE of N3 at a rain intensity of 56 mm h$^{-1}$ was 69.79 J m$^{-2}$ h$^{-1}$ and 42.32 J m$^{-2}$ h$^{-1}$ for N2.

**Table 1:** Results of calibration measurements of the three different nozzle types and rainfall intensities.

Nozzle-type	Intensity	Mean KE	KE under nozzle
	(mm h^−1^)	(J m^−2^ h^−1^)	(J m^−2^ h^−1^)
N1	18	11.38	92.57
N2	18	34.72	446.61
N2	28	93.36	1191.95
N3	28	77.62	337.01
N2	56	42.32	333.14
N3	56	69.79	276.96

### Salinity and temperature changes during rainfall in a turbulence-free water body

**Figure 2:**
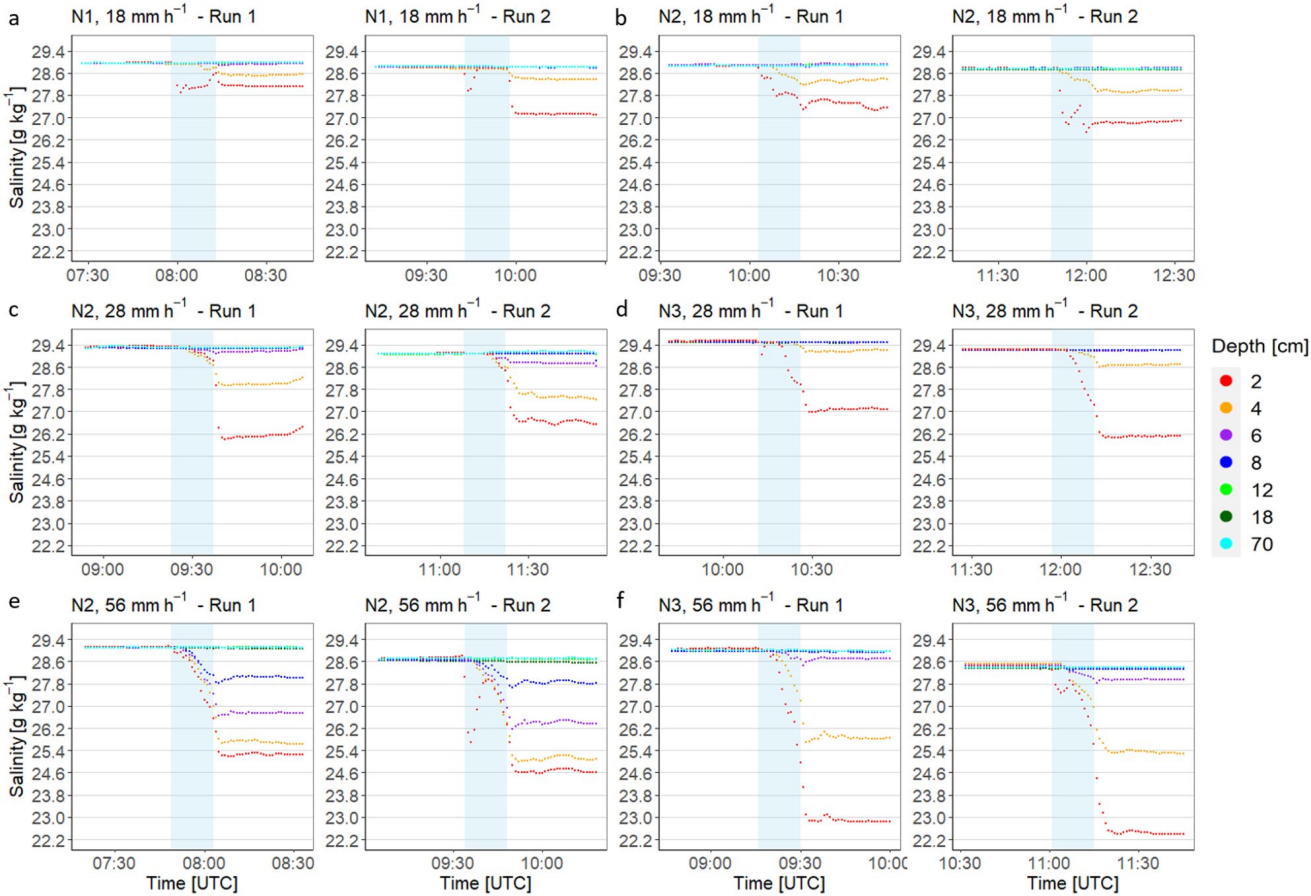
Time series of salinity during different rain scenarios with two repetitions (i.e., run 1 and 2) of experiments in a turbulence-free water body. It shows the lowest intensity of 18 mm h^−1^ (**a,b**), medium intensity of 28 mm h^−1^ (**c,d**), and highest intensity of 56 mm h^−1^ (**e,f**), and the different nozzle types with N1 (**a**), N2 (**b,c,e**), and N3 (**d,f**). The light blue rectangle indicates the 15-min rainfall period.

Salinity and temperature changes during the experiment in a turbulence-free water body with three different rain intensities of 18, 28 and 56 mm h^−1^ and three different nozzle types are shown in this section. Figure 2 shows the changes in salinity during the first rainfall experiments conducted in a turbulence-free water body. The difference in salinity before and after the artificial rainfall was calculated for each depth (see “Methods” section) and is described for each rainfall scenario in the following.

For all rainfall intensities and nozzle types, the SML was the most affected by freshwater input. At the lowest precipitation intensity of 18 mm h^−1^ and nozzles N1 and N2, the salinity in the upper 6 cm was affected by the rainfall, but not below. For example, with N1, which was characterized by the smallest outlet, the mean difference in salinity before and after rain was 1.22 ± 0.59 g kg^−1^, at a depth of 4 cm 0.39 ± 0.00 g kg^−1^, and at 6 cm 0.02 ± 0.01 g kg^−1^. Within the SML, the salinity decreased by a mean of 10.83 g kg^−1^ (see Table 2). The N2 nozzle, which produces a higher number of larger droplets, caused a lower mean salinity anomaly of 7.75 g kg^−1^ within the SML at the same rainfall intensity. At a nominal depth of 2 cm, the salinity changed by 1.67 ± 0.39 g^−1^ kg^−1^ min^−1^ and 0.69 ± 0.16 g^−1^ kg^−1^ min^−1^ at 4 cm during rainfall. At higher rainfall intensities of 28 mm h^−1^ and nozzle type N2, the salinity anomaly in the SML decreases further to 6.13 g kg^−1^ but increases at a nominal depth of 2 cm and 4 cm to 2.78 ± 0.50 g kg^−1^ and 1.39 ± 0.14 g kg^−1^ respectively, indicating accumulation of freshwater below the SML. With the same intensity and nozzle type N3, which produces fewer large drops and lower velocities, a shallower layer down to 4 cm was affected by the artificial rainfall, with a mean anomaly in salinity of 13.06 g kg^−1^ at the SML, 2.77 ± 0.46 g kg^−1^ at 2 cm, and 0.44 ± 0.17 g kg^−1^ at 4 cm.

The artificial rain of 56 mm h^−1^ generated with nozzle N2 caused a mean salinity anomaly of 5.42 g kg^−1^ at the SML, 3.92 ± 0.12 g kg^−1^ at 2 cm, 3.47 ± 0.12 g kg^−1^ at 4 cm, 2.31 ± 0.06 g kg^−1^ at 6 cm, and 0.96 ± 0.20 g kg^−1^ at 8 cm. With the same rainfall intensity but with nozzle N3, less rain water reached a depth of 8 cm, with a mean salinity anomaly of 14.18 g kg^−1^ in the SML, 6.02 ± 0.16 g kg^−1^ at 2 cm, 3.14 ± 0.02 g kg^−1^ at 4 cm, 0.40 ± 0.15 g kg^−1^ at 6 cm, and 0.05 ± 0.02 g kg^−1^ at 8 cm.

**Figure 3:**
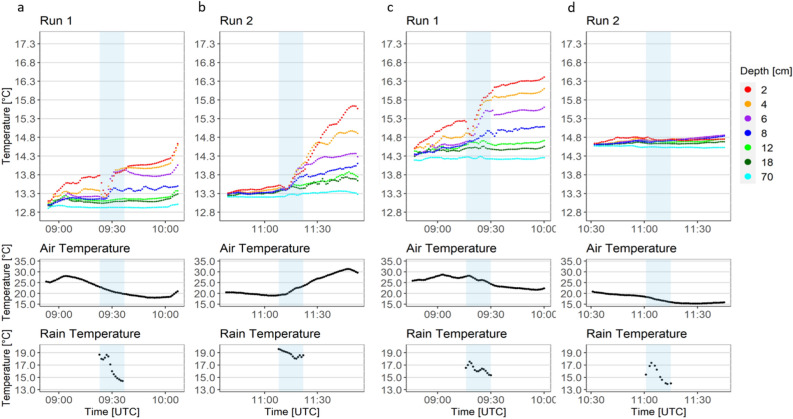
Time series of temperature changes during different rain scenarios with the first and second run of experiment one (turbulence-free water body). Shown are (**a,b**) nozzle type N2 with a medium intensity of 28 mm h$^{-1}$, and (**c,d**) N3 with the highest intensity of 56 mm h$^{-1}$. The light blue rectangle indicates the 15-min rainfall period. The first and second runs of the experiment represent replicates and were performed under the same conditions inside the tank.

The air temperature and artificial rain temperature were not controlled during the experiments (see “Methods” section). Temperature anomalies during the precipitation experiment using nozzle N2 (28 mm h^−1^) and N3 (56 mm h^−1^) are shown in Fig. 3. Additional temperature anomalies are shown in Fig. S2. The mean temperature for rain was 17.68 $^{\circ }\hbox {C}$ (N2, 28 mm h^−1^) and 15.88 $^{\circ }\hbox {C}$ (N3, 56 mm h^−1^). The temperatures within the upper 16 cm showed a strong dependence on air temperature and led to stratification at air temperatures above 20 $^{\circ }\hbox {C}$ (see Fig. 3a–c, Fig. S2). Together with the results of the second experiment (with turbulences, see “Methods” section) a significant dependence of the temperature at a nominal depth of 2 cm on the air temperature (Kruskal–Wallis test: p = 0.0044, chi-square = 205.01, df = 155, n = 224) was observed. However, the temperature of the artificial rain had no significant effect on the surface temperature anomalies at 2 cm (Kruskal–Wallis test: p = 0.2305, chi-square = 153.04, df = 141, n = 224). In Fig. 3a,b, the air temperature decreased during the experiment. In Fig. 3a, the air temperature rose from 25 to 29 $^{\circ }\hbox {C}$, and the surface temperature also increased in the upper 18 cm with increasing temperatures toward the surface, causing near-surface stratification. The peak in air temperature (09:05 UTC, Fig. 3a) coincided with water temperature peaks down to 18 cm depth, indicating a rapid response of the calm water body to the increase in air temperature triggered by a rise in solar radiation to 803 W m$^{-2}$. After 09:05 UTC, the air temperature declined, resulting in an immediate temperature drop of 0.1–0.2 $^{\circ }\hbox {C}$ between 6 and 18 cm, a rather constant temperature at 4 cm depth, and further increase in temperature at 2 cm nominal depth.

The temperature at the surface (0–4 cm) decreased during the first 4 min of the rainfall. The temperature at 2 cm decreased by 0.13 $^{\circ }\hbox {C}$ min^−1^, at 4 cm by 0.06 $^{\circ }\hbox {C}$ min^−1^, and at 6 cm by 0.01 $^{\circ }\hbox {C}$ min^−1^. Afterwards, the temperature at 4–6 cm increased and remained at similar levels for the remaining rainfall period. After the rainfall, the thermal stratification of the surface strengthened due to the absence of rain-induced mixing. In the second run with N2 (28 mm h^−1^) (Fig. 3b), the air temperature decreased slightly to 20 $^{\circ }\hbox {C}$. During the rainfall, the air temperature increased by 10 $^{\circ }\hbox {C}$ because of solar radiation on the closed canopy of the tank. Consequently, we observed a strong stratification in the upper surface layer, with the highest temperature change at 2 cm (2.25 $^{\circ }\hbox {C}$) and lowest at 18 cm (0.46 $^{\circ }\hbox {C}$).

Figure 2c (N3, 56 mm h$^{-1}$) shows a similar pattern in temperature compared to Fig. 2a (N2, 28 mm h$^{-1}$), but higher increase in water temperature due to higher intensity of warm rain. Figure 2d shows the second run of the experiment with nozzle type N3 (56 mm h$^{-1}$). The surface temperature increased to a depth of 18 cm with the layer at 2 cm warming first followed by a later increase at 18 cm. The air temperature decreased from 20.8 to 18.5 $^{\circ }\hbox {C}$ before the rain started. With the rainfall, the thermal stratification of the upper layers disappeared, and the air temperatures continued to decrease. No strong stratification built up as the air temperature was below 20 $^{\circ }\hbox {C}$.

**Table 2:** $\Delta$S$_{SML}$ in a turbulence-free and turbulence-mixed waterbody.

Nozzle-type	Intensity	Number of measurements	Turbulence-free/turbulence-mixed water body	Mean $\Delta$S$_{SML}$
	(mm h^−1^)			(g kg^−1^)
N1	18	3	Turbulence-free	10.83
N2	18	2	Turbulence-free	7.75
N2	28	2	Turbulence-free	8.41
N3	28	1	Turbulence-free	13.06
N2	56	2	Turbulence-free	5.42
N3	56	3	Turbulence-free	14.18
N1	18	1	Turbulence-mixed	2.51
N2	28	1	Turbulence-mixed	4.05
N3	56	1	Turbulence-mixed	6.17

SML samples were taken with the glass plate method before and after rainfall. $\Delta$S$_{SML}$ were calculated from the difference of SML salinity before and after rainfall.

### Salinity changes in a turbulent-mixed water body

A second experiment was performed with turbulent mixing in the water body, that is, a flow pump turned on with a power level of 1%, representing a mean turbulence kinetic energy (TKE) of 3.26 $\times$ 10$^{-4}$ m$^{-2}$ s$^{-2}$ at 14 cm depth. Additional observations without turbulences (pump stage: 0%) were conducted as reference (time series are shown in Fig. S3). Temperature anomalies ($\Delta$T = Temperature$_{0-18\, \mathrm{{cm}}}$ - Temperature$_{70\,\mathrm{{cm}}}$) are shown in Fig. S4.

**Figure 4:**
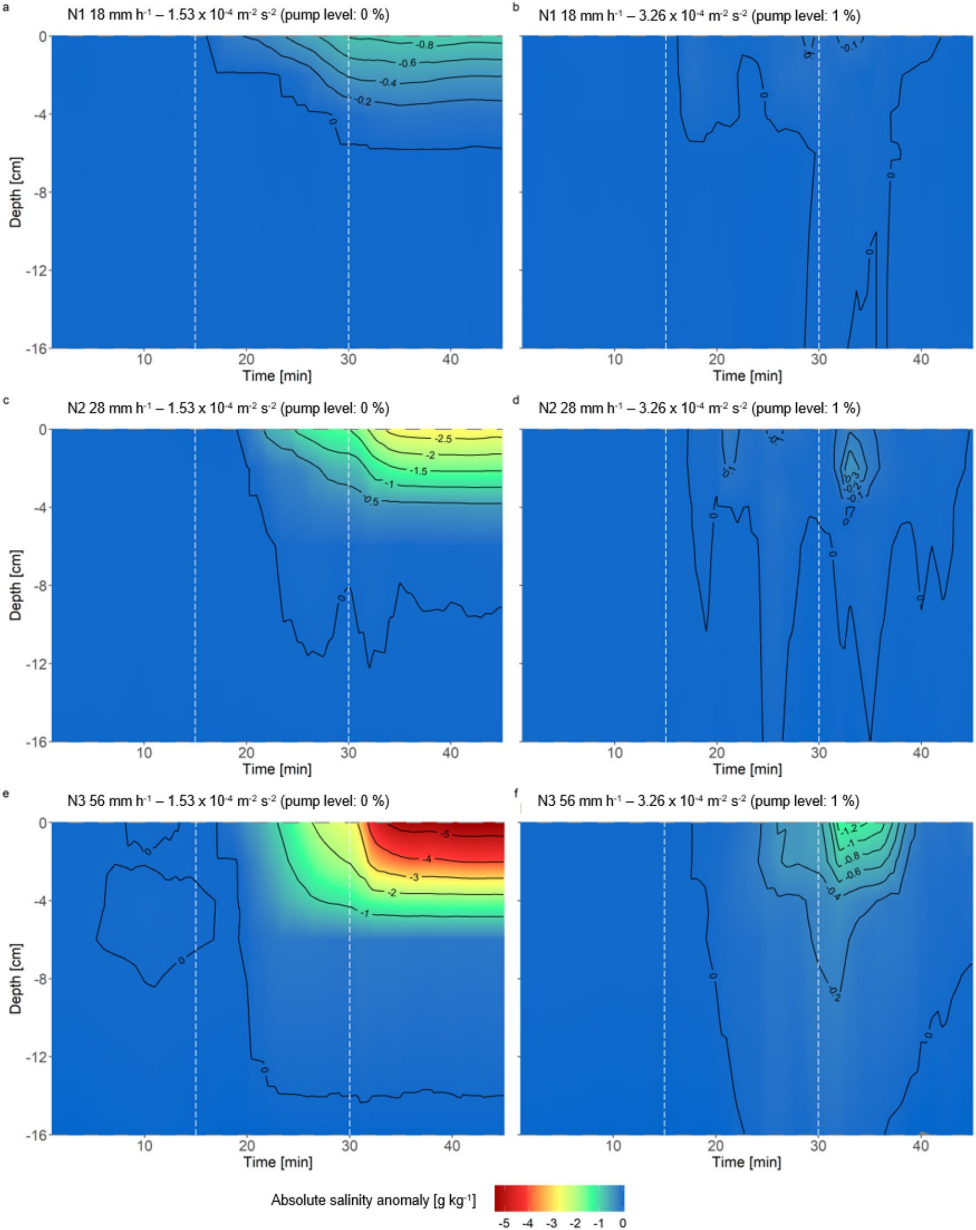
Contour plot of $\Delta$S at different depths during three rainfall scenarios in the second experiment with a turbulence-mixed water body. Shown are the lowest intensity of 18 mm h$^{-1}$ and N1 (**a,b**), the medium intensity of 28 mm h$^{-1}$ and N2 (**c,d**), and the highest intensity of 56 mm h$^{-1}$ and N3 (**e,f**) from a turbulent-free (**a,c,e**) and turbulence-mixed (**b,d,f**) waterbody with a pump level of 0% and 1%. The start and end times of the precipitation phase are indicated with a dashed line (minutes 16–30). The black isolines show the progression of equal salinities with time and depth.

Figure 4b,d,f show that the rain water was mixed rapidly with the seawater. Nevertheless, a freshwater lens can be seen at the surface after the end of the rain at all intensities with slightly negative $\Delta$D (Density$_{0-18\,\mathrm{{cm}}}$ - Density$_{70\,\mathrm{{cm}}}$, see “Methods” section) values (18 mm h$^{-1}$: maximum $\Delta$S = − 0.1 g kg$^{-1}$, 28 mm h$^{-1}$: maximum $\Delta$S = − 0.3 g kg$^{-1}$, and 56 mm h$^{-1}$: maximum $\Delta$S = − 1.2 g kg$^{-1}$) (see Fig. 4), that is, less saline water layer at the surface. This water layer also shows negative $\Delta$D values (see Fig. S5). The warmer freshwater lenses were observed with $\Delta$T of about 0.1 $^{\circ }\hbox {C}$ occurring for at least 5 min after the rainfall stopped (N3 and 56 mm h$^{-1}$) (Fig. S3f). At rainfall intensities of 18 mm h$^{-1}$ and 28 mm h$^{-1}$, the warmer lens disappeared at the end of the experiment. At the highest rain intensity, the warmer lens on the surface persisted for a longer period of time and was more pronounced.

Figure 4 shows the changes in $\Delta$S during the second experiment, comparing water bodies without and with turbulent flow. As shown in Fig. 2, the freshwater from the rainfall caused stratification and formed a freshwater lens in the upper 10 cm in a waterbody free of turbulent mixing. As the rainfall intensity increased, the influence of artificial rain was more pronounced. At the lowest rainfall intensity of 18 mm h$^{-1}$, salinity was influenced by a depth between 4 and 8 cm, with a maximum $\Delta$S of − 0.92 g kg$^{-1}$. At a rainfall intensity of 28 mm h$^{-1}$, salinity decreased to a depth of 12 cm with a maximum $\Delta$S of 2.80 g kg$^{-1}$. At the highest intensity of 56 mm h$^{-1}$, salinity decreased to a depth of 12–16 cm, with a maximum $\Delta$S of 5.53 g kg$^{-1}$. However, clear stratification did not occur until the rain had stopped, which was about 4 to 5 min afterward (Fig. 4a,c,e). The stratification occurring after rainfall was also evident in the density anomalies (Fig. S5), where $\Delta$D was lower closer to the surface.

Furthermore, salinity changed faster during the rain phase with increasing rainfall intensity in the turbulence-free waterbody. At the surface (nominal 2 cm), $\Delta$S changed 0.05 g kg min$^{-1}$, with the lowest rainfall intensity of 18 mm h$^{-1}$. At medium rainfall intensity (28 mm h$^{-1}$), $\Delta$S changed twice as quickly with 0.1 g kg min$^{-1}$. At the highest intensity of 56 mm h$^{-1}$, salinity changed with 0.17 g kg min$^{-1}$, that is, thrice compared with 18 mm h$^{-1}$ (see Fig. 3a,c,e). The salinity of the SML changed with the onset of rainfall, and the extent of the change increased with increasing rainfall intensity. At rainfall intensities of 18 mm h$^{-1}$, 28 mm h$^{-1}$, and 56 mm h$^{-1}$, $\Delta$S$_{SML}$ was 2.51 g kg$^{-1}$, 4.05 g kg$^{-1}$, and 6.14 g kg$^{-1}$, respectively (see Table 2). This pattern approximately follows the respective increase in rainfall intensity.

Turbulent mixing was performed throughout the rainfall experiment, with a mean TKE of 3.26 $\times$ 10$^{-4}$ m$^{-2}$ s$^{-2}$ (Fig. 4b,d,f). At all rainfall intensities, a fresher lens appeared very close to the surface after the rain ended. The freshwater lens was most pronounced at 56 mm h$^{-1}$ and was still present 15 min after the rain, with a maximum salinity anomaly of − 1.32 g kg$^{-1}$.

## Discussion

This study focused on the changes in salinity at the surface. It showed that in a turbulence-free water body, changes in the salinity of up to 6.02 g kg$^{-1}$ during intense rainfall (i.e., 56 mm h$^{-1}$) directly at the surface (0–4 cm). A rainfall intensity of 56 mm h$^{-1}$ corresponds to a heavy rain according to the World Meteorological Organization (WMO) (see Fig. 3). Salinity anomalies in the upper centimeters (relative to prerainfall salinity) increased with increasing rain intensity in the turbulence-mixed water body (see Fig. 4). This indicates that the magnitude of salinity anomalies rose with increasing rain intensity and that temperature and salinity anomalies developed more rapidly at higher intensities. The absence of wind has been shown to be one of the most important factors in the formation of freshwater lenses^28,32,33^, but the interplay between wind and precipitation is not yet fully understood. In our experiments, we consciously excluded some external forces, such as wind-driven mixing. The experimental approach with different nozzle types and flow rates was able to generate precipitates with different droplet properties and intensities. However, artificial rain differs from natural rain, which often reaches even larger diameters. Still, our results have confirmed in situ observations during very low wind conditions, showing that surface freshening is proportional to rainfall intensity (see Fig. S6) and that higher intensities are associated with faster changes in salinity^25,26,34^.

Reverdin et al.^35^ described a heavy precipitation event with a maximum rainfall rate of 37 mm h$^{-1}$ over 90 min under low wind conditions (in the range of 1.07–3.68 m s$^{-1}$), showing a salinity change of up to 9.5 g kg$^{-1}$ at a depth of 5 cm with significant stratification of the first upper 23 cm of the sea surface. Our experiments reached comparable results with a maximum salinity difference of 6.02 g kg$^{-1}$ in 0–4 cm depths and with a higher rainfall intensity but shorter rain period. With wind conditions of less than 1 m s$^{-1}$, the difference (54–0 cm) of salinity and temperature were observed to reach 10.5 g kg$^{-1}$ and 2 $^{\circ }\hbox {C}$ with a rainfall intensity of more than 20 mm h$^{-1}$ in a study by Iyer and Drushka^33^. Using the smallest nozzle in the turbulence-free experiment caused a similar mean salinity change in the SML of 10.83 g kg$^{-1}$ (see Table 2).

However, our first experiment in the turbulence-free water body and using different nozzle types shows that not only did the rain intensity have an influence on the distribution of freshwater during rain events, but also the droplet properties play an important role. The impact of droplets hitting the sea surface can best be described by the KE, which is calculated by the velocity and size of the raindrops. This parameter has been widely used to describe soil degradation because of rainfall^36,37^. The results are presented in Table 1 and show that, when comparing the different types of nozzles at the same level of rainfall intensity, different KE values were generated, which influenced the penetration depths. In the turbulence-free water body and with the lowest rainfall intensity of 18 mm h$^{-1}$, the KE of N2 was higher than that of N1, and the penetration depth was deeper because the depth of 4 cm was more affected by the rainwater (Fig. 3). In addition, the salinity of the SML decreased less with N2 ($\Delta$S$_{SML}$ = 7.75 g kg$^{-1}$, KE = 34.72 J m$^{-2}$ h$^{-1}$) compared with N1 ($\Delta$S$_{SML}$ = 10.83 g kg$^{-1}$, KE = 11.38 J m$^{-2}$ h$^{-1}$), indicating that the droplets remained more at the surface with lower KE. We made similar observations at the intermediate intensity of 28 mm h$^{-1}$, where N3 generated lower KE ($\Delta$S$_{SML}$ = 13.06 g kg$^{-1}$, KE = 77.62 J m$^{-2}$ h$^{-1}$) than N2 ($\Delta$S$_{SML}$ = 8.61 g kg$^{-1}$, KE = 93.36 J m$^{-2}$ h$^{-1}$). In the case of using N3, we observed a higher salinity anomaly in the SML and a shallower penetration depth of 6 cm compared with N2 with the same rainfall intensity. At the highest rainfall intensity of 56 mm h$^{-1}$, the SML salinity anomaly observed with N3 ($\Delta$S$_{SML}$ = 14.18 g kg$^{-1}$, KE = 69.79 m$^{-2}$ s$^{-2}$) was significantly higher than the anomaly observed with N2 ($\Delta$S$_{SML}$ = 5.42 g kg$^{-1}$, KE = 42.32 m$^{-2}$ s$^{-2}$), despite the fact that N3 had a higher mean KE. KE values were determined by the optical disdrometer prior to the experiments and placed directly above the water surface. Conductivity, temperature and depth (CTD) sensors were mounted afterwards at the same locations for vertical profiling of temperature and salinity. We observed a significantly higher KE of N2 compared with N3 directly below the nozzle (see Table 1), which explains why freshwater penetrates deeper with the nozzle N2 compared with N3 at the same rainfall intensity. Figure S1 shows that the distribution of rain droplets from N2 and N3 were different. N2 produced a higher number of intermediate droplet sizes and that had higher velocities.

**Figure 5:**
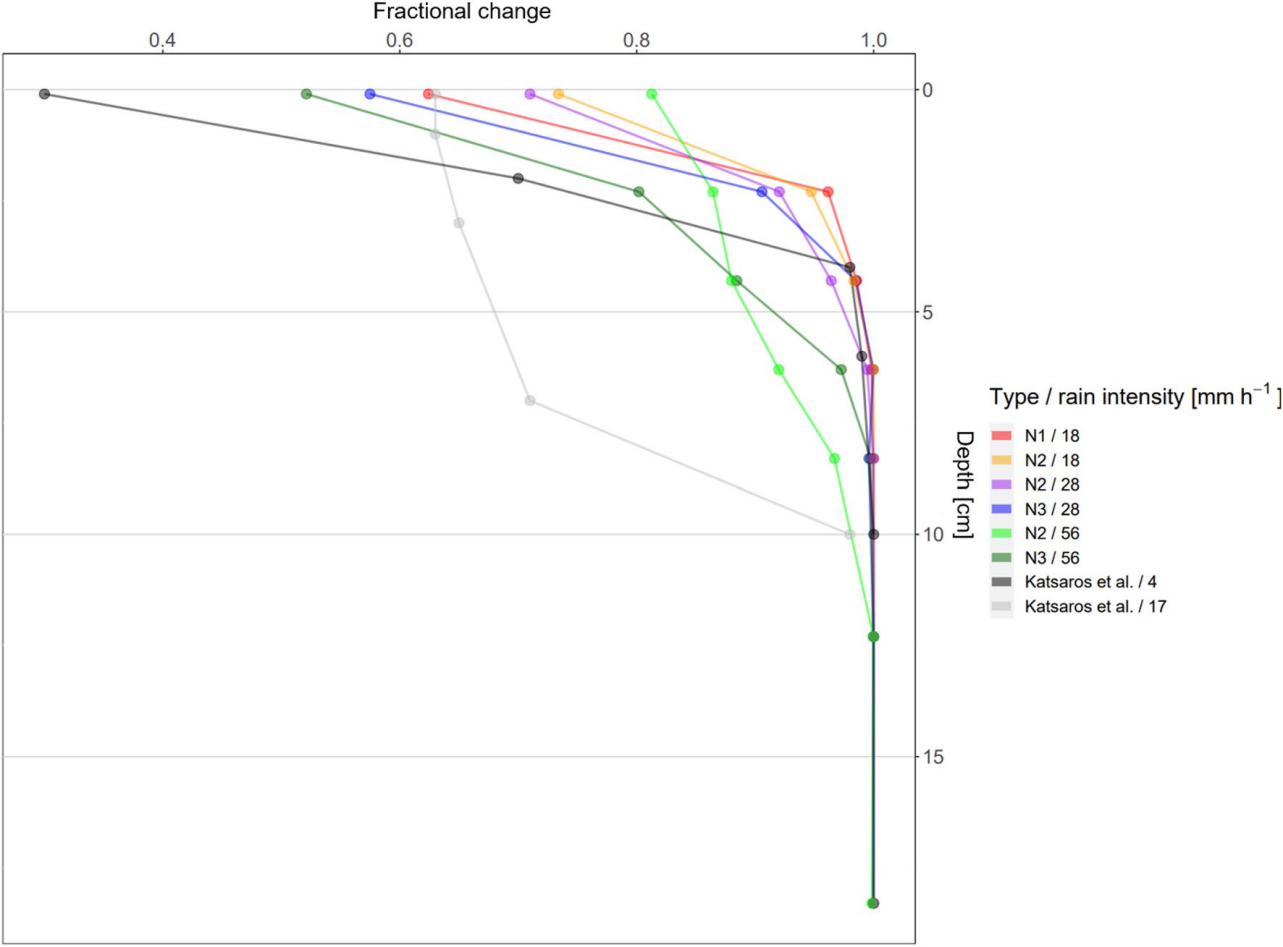
Mean fractional change of salt content in the surface waters in the turbulence-free water body with different nozzle types and rain intensities. Additional the fractional change of the artifical rain experiments of Katsaros and Buettner^21^ are shown as dashed lines with two different rain intensities of about 4 mm h$^{-1}$ with a droplet size of 1.2 mm and a rain intensity of 17 mm h$^{-1}$ with a droplet size of about 3 mm. A value of 1 indicates undiluted sea water and 0 pure rain water.

Katsaros and Buettner^21^ conducted one of the few experiments with artificial rainfall and two different droplet sizes, measuring the conductivity and temperature at different depths in a turbulance-free tank. They conducted their experiments with two different rain intensities of about 4 and 17 mm h$^{-1}$ and one droplet size for each intensity, of 1.2 mm for 4 mm h$^{-1}$ and 3 mm for 17 mm h$^{-1}$. A comparison of their result and ours are shown in Fig. 5 and the calculation of the fractional change is described in the Supplementary Material. The results show that smaller droplets together with a low rain intensity produced a larger salinity anomaly at the surface (compared with larger droplets and higher rain intensity), but the magnitude of these anomalies decreased more rapidly with depth than for larger droplets. Larger droplets generally have a higher KE, creating a vortex ring that penetrates downward into the sea surface^22,38^. The penetrating droplets additionally caused turbulence in the near-surface water^18,19^, indicating that, in our experiments in turbulence-free waters, distinct stratification developed only after the precipitation phase and that the stratification of the temperature was removed with the onset of rain. The TKE values at a depth of 14 cm have confirmed that the TKE was higher during the rainy phase than during the nonrainy phase (rainy phase = 0.003 ± 0.001 m$^{-2}$ s$^{-2}$ , nonrainy phase = 0.001 ± 0.001 m$^{-2}$ s$^{-2}$). Interestingly, the intensity of 17 mm h$^{-1}$ from the Katsaros and Buettner^21^ experiment caused the same fractional change as in our results N1 and a rain intensity of 18 mm h$^{-1}$ at the surface. The larger droplet sizes used by Katsaros and Buettner^21^ in their experiments penetrated deeper into the surface and caused a high fractional change in salinity to a depth of about 7 cm. This observation shows that droplet size has an influence on the distribution of freshwater in the surface ocean in addition to rainfall intensity. In our experiment, we did not detect very high fractional changes at depths greater than 4 cm, probably because we could not achieve droplet sizes larger than 1.5 mm with our approach. The rain intensity of 56 mm h$^{-1}$ of N2 and N3 showed that the fractional change was slightly higher between 4 and 8 cm depth, which could be caused by a larger amount of larger droplets with higher velocity that penetrated deeper into the surface.

Overall, comparing data from this study (N2/18, N2/28, N2/56) and by Katsaros and Buettner^21^ (Fig. 5), distinct profiles are driven by rainfall intensities. The higher the rainfall intensity and the larger the droplets, the more freshwater are accumulating below the nominal depth of 2 cm (0–4 cm). However, we found that analysis of droplet distribution is important. For example nozzle N3 generated a larger fraction of smaller droplets (0–0.125 mm) and larger droplets (> 0.375 mm) with an intensity of 56 mm h$^{-1}$ compared to an intensity of 28 mm h$^{-1}$ (Fig. 1). The larger droplets at an intensity of 56 mm h$^{-1}$ caused higher accumulation of freshwater below the nominal depth of 2 cm, but did not complete interfere with the accumulate of freshwater at the surface (0 cm in Fig. 5) due to the larger fraction of smaller droplets. It illustrates the complexity and the need of further systematic studies in the future to develop an empirical parameterization for the fate of freshwater in the upper ocean.

In the turbulence-free waterbody, the stratification of the surface persisted for at least 30 min after the rainfall had stopped. The experiments conducted by Katsaros and Buettner^21^ confirmed the evolution of stratification, which persited for at least 2 h. This shows that the freshwater lens would properly resist for many hours without any turbulence in the water body of our tank, i.e. without wind-induced mixing and gravity flows^39^. In situ, freshwater lenses were observed to resist for more than 10 h with ongoing rain events^33^. Typically freshwater lenses can last several hours under low wind conditions^27,40^. The freshwater lowers the density, hence inhibiting vertical mixing^18,32,35^.

The second experiment, with a mixed water body, confirmed that turbulence in the water body reduces the formation of freshwater lenses; that is, smaller anomalies were observed. Likewise, the residence time of fresher water masses at the surface after a rain event was shortened so that, for each of the three rain intensities applied, the fresher water layer at the surface had disappeared after 15 min and the water mass had mixed with the layers below. Our results confirm the observations of ten Doeschate et al.^41^, who observed that vertical temperature and salinity anomalies are best described by total rain, i.e., rainfall intensity relative to time. They also noted that wind speeds and associated turbulence play a role in the formation of freshwater lenses. With the flow pump at 1%, we obtained a mean TKE of 3.26 $\times$ 10$^{-4}$ m$^{-2}$ s$^{-2}$ near the surface at a depth of 14 cm. Here, 12.17% of the freshwater from the artificial rain remained in the first 2 cm of the surface during the 15-min postrain phase within the turbulence-free waterbody (see Fig. S4). Compared with the turbulence-mixed water body, only 2.33% remained in the surface layer. In the artificial rainfall experiments from Katsaros and Buettner^21^, the change in salinity between 2 and 4 cm depth was three times larger compared with our experiments, which may be because their experimental set up used two different defined droplet sizes of 1.2 mm and 3 mm, which are larger compared with our generated droplet sizes. With higher intensity, the amount of freshwater also increased, and at the mean intensity of 28 mm h$^{-1}$, 29.07% remained in the top 2 cm in the turbulence-free water body and 2.81% in the turbulence-mixed water body. Most of the freshwater remained at the surface with the highest intensity of 56 mm h$^{-1}$ with 32.01% and 3.77% in the turbulence-mixed water body. This confirms that the influence on surface salinity was very dependent on rain intensity and that a typical coastal regime for turbulence^42^ within the surface layer mixed up to 90% of freshwater rapidly into the mixed layer or was lost by wind-driven enhancement of evaporation.

The results on the surface temperature show that in the first experiments (turbulence-free water tank) it was dependent on the ambient air temperature, which could not be controlled and therefore varied between the different runs. Warm rain occurs typically in the presence of vigorous convective clouds due to the collision and coalescence of rain droplets. Warm rain occurs more typically over the ocean, as aerosols over land suppresses the process of coalescence^31^. In this context, the artificial rain in our study refers to rainfalls more typical for thunderstorms over the ocean. As solar radiation is absorbed within the upper ocean, heat becomes confined within the upper meters, giving rise to a buoyant, highly stratified warm layer that correlates with an increase in SST. We have observed such warming prior the rain events (Fig. 2) and such warming decreased with increasing depths. With the onset of rain, mixing of the initial stratification occurred within the first few minutes of the rain. Since the simulated rain was warmer than the water, typically for thunderstorms^31^, additional heat was added by the rain as indicated by the increase of SST during the rain. After the rain, the buoyant and stratified surface layer continued to absorbed heat. That was particular true in the case of increasing air temperature (Fig. 2b). Heat flux measurements were not conducted, but in the first three cases (Fig. 2a–c), the sensible heat flux was reduced due to the higher air temperature and increasing SST. On the other hand, the latent heat flux likely increased due to the increased SST with the assumption of constant humidity and air flow. As long-wave heat flux depends on SST according to the Stefan-Boltzmann law, the fourth term of the net heat flux increased with the assumption of a constant emissivity of the ocean surface.

Our simulation of artificial rain and thus the insights gained relate to thunderstorms due to the warm rain. The limitation is that in our simulations the proportion of larger raindrops is smaller than over the ocean. This demonstrates the difficulty of generating artificial rain with specific properties. Nevertheless, our study shows the fundamental processes of how rainwater is distributed in the surface ocean (Fig. 5). Additional process-oriented mesocosm studies and more comprehensive in situ data sets^11,12^ are required to parameterize salinity anomalies in the SML and near-surface layer to rain falls, i.e. to the fate of freshwater in the ocean.

## Conclusion

Our experiments with artificial rain contributed to a better mechanistic and quantitative understanding of the effects of rainfall on sea surface salinity, which serves as important key indicator of the oceanic hydrological cycle. We were not primarily aiming at reproducing in situ events, because these cannot be simulated realistically, but at gaining fundamental knowledge about the depths and the speed at which freshwater causes anomalies at certain intensities. In our facility, we excluded the influence of wind, thus minimizing evaporation to gain a better understanding of the role of rainfall intensity and droplet properties in the distribution of freshwater lenses. We have shown that the strength of rainfall intensity had the greatest influence on the anomalies in surface salinity and on how rapidly the anomalies occurred. The KE with which the drops hit the surface influenced the amount of freshwater remaining in the SML and how deep the freshwater has penetrated the surface. We were able to show in our study that thermal stratification can be partially removed by the mixing effect of rain. Turbulence inside the waterbody mixed the freshwater rapidly with the bulk seawater mass, but such turbulent mixing was limited, and complete mixing took longer with a less saline sea surface after the rainfall event. In the presence of turbulence in the water body, rainfall intensity was still an important factor determining salinity anomalies at the surface. We show that in a turbulence-free and mixed water body, the salinity anomalies at the SML were the highest. The results provide new insights into how freshwater is distributed over the ocean surface as a function of rainfall intensity and droplet properties. Our studies underline the importance of the SML, which is as boundary layer most affected by the freshwater cycle^11,12^. For further studies, the complex interaction of wind-driven mixing, advection, and evaporation needs to be resolved, both under controlled and in situ conditions. This would give us the opportunity to obtain a better understanding of freshwater fluxes, that is, evaporation minus precipitation, between the ocean and atmosphere, and, therefore, on the oceanic hydrological cycle. Overall, a mechanistic understanding and in situ observation are crucial for a solid foundation toward future model efforts of the oceanic hydrological cycle–a poorly understood component of the global hydrological cycle.

## Methods

### General experimental set-up

**Figure 6:**
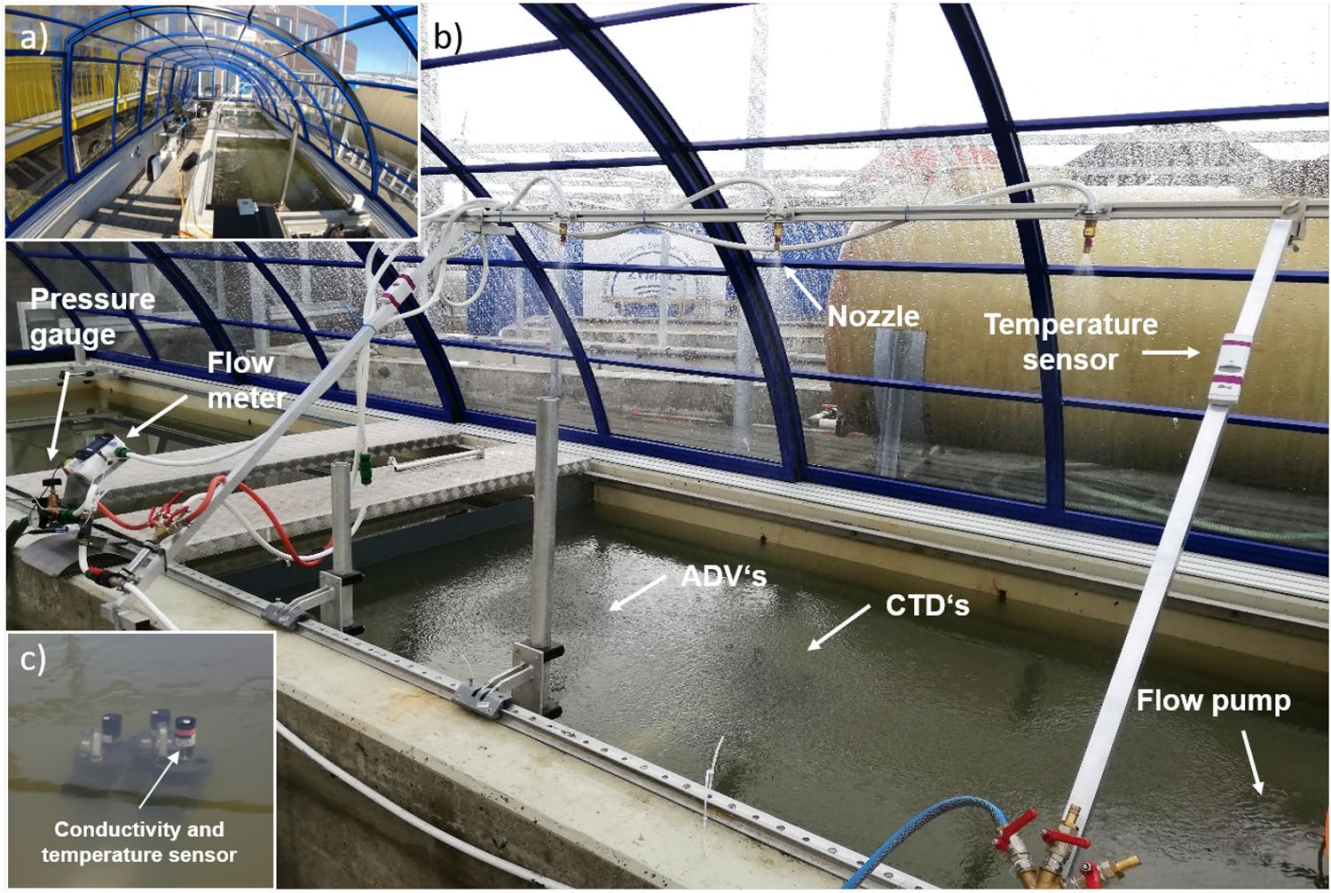
(**a**) SURF facility with the entire length of the covered tank and (**b**) tank of SURF with the experimental set up, including the artificial rainfall unit with the pressure gauge and flow meter to regulate the rain intensity, the acoustic doppler velocimeter (ADV), and the conductivity temperature depth sensors (CTD) placed inside the tank on a position below the nozzle. The positions of the flow pump and temperature sensors are also shown. (**c**) The CTDs in a vertical upward position with the conductivity and temperature sensor close to the surface.

The experiments were conducted in the Sea sURface Facility (SURF), which is located at the Institute for Chemistry and Biology of the Marine Environment (ICBM; Wilhelmshaven, Germany). SURF consists of a concrete tank (8 m $\times$ 2 m $\times$ 0.8 m), a retractable canopy, seawater pipeline, and large-scale filtration units (Fig. 6). For the experiment, 5100 L untreated seawater was pumped from Jade Bay (North Sea) into the tank and sigma-t values were approx. 21–22 kg m$^{-3}$. For the mesocosm experiments, we reduced the size of the tank to 3.2 $\times$ 2 m by installing a partition wall. The water level in the tank was 0.8 m. The facility was covered with a canopy made of transparent polycarbonate, which allowed sunlight to pass through but protected the facility from other external influences such as wind or natural precipitation. Above the water surface, a rain simulation system was installed at a height of 1.33 m. Tap water flowed through hoses to a pressure regulator and flow meter (Flowmax 42i, MIB GmbH, Germany) that then went into three or four nozzles that distributed the water evenly as rain over the entire tank^43^. We simulated rain intensities of 18 mm h$^{-1}$, 28 mm h$^{-1}$, and 56 mm h$^{-1}$, which required a flow rate of 0.5 L min$^{-1}$, 1 L min$^{-1}$, and 2 L min$^{-1}$, respectively. The flow rate was set with the pressure regulator and measured with the flow meter. We measured and recorded the temperature of the artificial rainwater every 30 seconds with a temperature probe ($\hbox {WTW}^{\textrm{TM}}$ $\hbox {MultiLine}^{\textrm{TM}}$ 3420, Xylem Inc., USA) that was installed between the pressure gauge and flow meter. Natural rain often scales with the intensity of precipitation^44^. This is why three different types of nozzles (1/8” GG1, GG2, and GG3; Spraying Systems Germany GmbH, Germany), hereinafter referred to as N1, N2, and N3, were used to produce different droplet characteristics. The nozzles differed in outlet size. N1 had an outlet of 0.79 mm in diameter, N2 an outlet of 1.2 mm, and N3 an outlet of 1.5 mm. At higher flow rates, more water flowed through the nozzles and the pressure also increased, so the droplets could be sprayed more finely and with higher fall velocities.

Tap water was distributed to several nozzles of the same type. Four nozzles spaced 60 cm apart were used to simulate the lowest rainfall rate of 18 mm h$^{-1}$. For the other two intensities of 28 and 56 mm h$^{-1}$, three nozzles spaced 80 cm apart were sufficient to cover the entire area. All hoses connected to the nozzles had the same length for equal flow and rain intensities among the nozzles used in the individual experiments. The flow rate was constantly monitored at one nozzle during the experiments. The flowmeter provided the total volume of water that flowed through the system, and these values were always noted before and after the rain. The difference between the total amount of water that flowed through the flowmeter before and after the artificial rain gave the amount of water that flowed through the nozzles during the experiment. Simulated raindrops as well seawater characteristics are more representative for mid-latitudes than of tropical regions. The air temperature and humidity were measured inside SURF at a 1-minute interval using a weather station (Klimalogg Pro, TFA Dostmann, Germany). Solar radiation was measured outside the facility with a weather station (TDL14K, Thies Clima, Germany).

### Calibration of the artificial rainfall

Before the actual experiments, we calibrated the different nozzle types and rain intensities. A laser-based distrometer (Model no. 5.4110.10.xxx, Thies Clima, Germany) was installed directly above the water surface to measure droplet characteristics. The laser beam covered an area of 45.6 cm$^{2}$ and categorized raindrops in 22 sizes and 20 velocities. For each nozzle type and rain intensity, measurements of droplet characteristics were repeated twice at 15 different positions under the artificial rain produced by the nozzles (Fig. S7). The final velocity requires a certain drop height to be reached, and this height is limited in our experiments due to the canopy. However, the disdrometer used measures the actual fall velocity, from which we calculated the kinematic energy (KE) of the different drops according to Lassu et al.^37^:

1 $$\begin{aligned} KE = \frac{\pi }{12} \times \frac{1}{6^{10}} \times \frac{3600}{t} + \frac{1}{A} \times \sum _{i=t}^{20} n_{i} \times D_{i}^{3} \times (V_{D_{i}}), \end{aligned}$$

where ‘*A*’ is the measured surface of 45.6 cm$^{-2}$, ‘$n_{i}$’ the number of drops of diameter ‘$D_{i}$’, ‘$V_{D_{i}}$’ the fall velocity of drop with diameter ‘$D_{i}$’, and ‘*t*’ 60 s, here representing the time of measurement. The mean kinetic energy of the whole area of the calibration design and kinetic energy of the position directly below the nozzle (see Fig. S7) are shown in Table 1.

### The first experiment: turbulent-free conditions

In the first experiments, the fate of rainwater was investigated in a stagnant water body, that is, without any turbulence generated. Seven CTD (conductivity, temperature, and depth) sensors (Ocean Seven 310, Idronaut, Italy), with an accuracy of 0.0015 mS cm$^{-1}$ and 0.0015 $^{\circ }\hbox {C}$, were mounted in the tank vertically upward (see Fig. S9, which shows a comparison between vertically and horizontally mounted CTDs in a test run) at depths between 0 and 16 cm directly below one of the nozzles (see Fig. 6). The conductivity cell had a length of 4 cm, that is, the nominal depth of 2 cm covered 0–4 cm, 4 cm: 2–6 cm, 6 cm: 4–8 cm, 8 cm: 6–10 cm, 12 cm: 10–14, and 18 cm: 16–20 cm depths. With this setup, about the first 20 cm of the surface was completely resolved by conductivity and temperature measurements. An additional CTD (48M; Sea and Sun Technology, Germany), with an accuracy of 0.002 mS cm$^{-1}$ and 0.002 $^{\circ }\hbox {C}$, was placed at a depth of 70 cm in a horizontal position. The nozzles spray in a circular rather than a square pattern, and the outer corners of the water surfaces are likely less affected by the rain. However, the CTDs are mounted in the center of the spray field and detect the predominant influence of rain on temperature and salinity.

Before the experiment, the water in the tank was completely mixed for 15 min with a flow pump (AFC400IPU, Abyzz, Germany). The water was then allowed to settle for 15 min. After that, the experiment started with a prephase without rain for 30 min. Following this, rain was simulated for 15 min with selected combinations of nozzle types and flow rates (Table 1). After the artificial rain period, temperature and conductivity measurements were continued for another 30 min. This was repeated twice for each combination of intensity and nozzle type, as shown in Table 1. Before and after the rain period, samples from the SML were collected with a skimming technique using a glass plate^45^. Because of surface tension, the SML adhered to the glass with slow vertical withdrawal of the plate from the water. Adhering SML was collected with a wiper into a glass bottle according to the best practice guide for the collection of the SML^46^. The conductivity of the SML samples was measured with a salinometer (OPTIMARE Precision Salinometer), and the absolute salinity was calculated according to TEOS-10.

### The second experiment: turbulent-mixed conditions

The second experiment was conducted with a very similar setup as the first experiment, but turbulent regimes inside the water body were generated by the flow pump. For measuring TKE, two single acoustic doppler velocimeter (ADV) sensors (Vector, NORTEK, Norway) were mounted in the tank at 14 and 44 cm depths and below one of the nozzles. The experiments were conducted at three different power settings of the flow pump (no-flow: 0%, first-level-flow: 0.5%, and second-level-flow: 1%), generating the following TKE regimes. The power level of 0%, that is, flow pump was off, and a mean TKE of 1.53 $\times$ 10$^{-4}$ ± 1.26 $\times$ 10$^{-4}$ m$^{-2}$ s$^{-2}$ at 14 cm and 2.95 $\times$ 10$^{-5}$ ± 1.12 $\times$ 10$^{-5}$ m$^{-2}$ s$^{-2}$ at 44 cm were recorded. The power setting of 0.5% caused a mean TKE of 3.37 $\times$ 10$^{-4}$ ± 1.00 $\times$ 10$^{-4}$ m$^{-2}$ s$^{-2}$ at 14 cm and 2.14 $\times$ 10$^{-4}$ ± 6.31 $\times$ 10$^{-5}$ m$^{-2}$ s$^{-2}$ at 44 cm, and the power setting of 1% had a mean TKE of 3.26 $\times$ 10$^{-4}$ ± 7.82 $\times$ 10$^{-5}$ m$^{-2}$ s$^{-2}$ at 14 cm and 2.33 $\times$ 10$^{-4}$ ± 5.71 $\times$ 10$^{-5}$ m$^{-2}$ s$^{-2}$ at 44 cm depth. The 0.5% and 1 $\%$ power setting generated similar TKE levels comparable to TKE measurements taken in coastal regions, such as the Jade Bay^42^. For this reason, we provided data from the 1% power setting in the results section, and data from the 0.5% powering setting can be found in the supplementary material (Fig. S8). Before starting the second experiment, the water body was completely mixed for 15 min. After that, the water settled for 15 min. Subsequently, the experiment started with no turbulence, a 0.5% or 1% pump level for a total time of 45 min with the following procedure: no rainfall for 15 min, then artificial rain with a constant intensity for 15 min, and, finally, an additional period of 15 min without rain. Before and after the rain period, samples of the SML were taken to determine salinity. Three rain scenarios with N1 18 mm h$^{-1}$, N2 28 mm h$^{-1}$, and N3 56 mm h$^{-1}$ were simulated (Table 1). Temperature and conductivity were measured at the same depths as in the first experiment, except that the 10 cm depth was not measured because of technical issues with a CTD.

The TKE was calculated from the 16 Hz velocity measurements of each ADV for bins of 15 min using the Python dolfyn toolbox by Levi Kilcher, as follows:

2 $$\begin{aligned} E = \frac{1}{2} (\overline{u'^{2}} + \overline{v'^{2}} + \overline{w'^{2}}), \end{aligned}$$

where ‘*u*’, ‘*v*’, and ‘*w*’ represent the velocity of the 3D velocity components in the form of their variances, with the prime indicating the fluctuation from the mean and the overbar the mean value. This allowed us to examine the turbulence levels before, during, and after the rain events at the two different depths of the water column.

### Data analysis and statistical tests

To avoid a bias between the CTD measurements, the salinity was corrected with the anomalies between the CTDs observed during the complete mixing between the experiments. The anomalies were in the range of 0.19 to 0.53 g kg$^{-1}$. The anomalies between the deepest salinity and temperature measurement in the tank, at 70 cm depth, and the five to six different depths closer to the surface were calculated ($\Delta$S = Salinity$_{0-18\,\mathrm{{cm}}}$ − Salinity$_{70\,\mathrm{{cm}}}$, $\Delta$T = Temperature$_{0-18cm}$ − Temperature$_{70cm}$, $\Delta$D = Density$_{0-18\,\mathrm{{cm}}}$ − Density$_{70\,\mathrm{{cm}}}$). Anomalies were also calculated from the measured salinities of the SML samples before and after the rainfalls: $\Delta$S$_{SML}$ = Salinity$_{before}$ − Salinity$_{after}$. Contour plots were computed with the R (Version 4.2.1), “interp” package , and the resolution of x and y was set to 300. The statistical significance of the influence of air temperature and rain temperature on the temperature anomalies of 2 cm depth was determined using the Kruskal–Wallis test. The Kruskal–Wallis test is a rank-based non-parametric test used to determine whether there are statistically significant differences between two or more groups of an independent for a continuous dependent variable. The results were considered significant when p $\le$ 0.05, with a 95% confidence level. The Chi$^{2}$ represents the sum of squared deviations for an expected pattern. The degrees of freedom (df) indicate the quantity of independent values.

### Supplementary Information

Supplementary Information.

## Data Availability

All the data generated or analyzed during this study is available in this published article and on PANGAEA^47^.
