# Supplementary material for: The impact of rainfall on the sea surface salinity: a mesocosm study
Source: Sci Rep. 2024 Mar 16;14:6353. doi: 10.1038/s41598-024-56915-4 (PMC11343853; doi:10.1038/s41598-024-56915-4)
Supplement: Supplementary file 1 — Supplementary Information. [file 41598_2024_56915_MOESM1_ESM.pdf]

Supporting information for "The impact of rainfall on the sea surface salinity – a mesocosm study"

Lisa Gassen[1,*], Leonie Esters[2,3], Mariana Ribas-Ribas[1], and Oliver Wurl[1]

[1] *Center for Marine Sensors (ZfMarS), Institute for Chemistry and Biology of the Marine Environment (ICBM), School of Mathematics and Science, Carl von Ossietzky Universität Oldenburg, Ammerländer Heerstraße 114-118, 26129 Oldenburg, Germany*

[2] *Institute for Geosciences, University of Bonn, Bonn, 53115, Germany*

[3] *Department of Earth Science, LUVAL, Uppsala University, Uppsala, 75236, Sweden*

[*] *lisa.gassen@uni-oldenburg.de*

March 6, 2024

Supporting information

Calculation of freshwater proportion at the surface

We used the following equation to calculate the freshwater proportion (FP) staying within the surface layer (upper 2 cm) after the rainfall of the second experiment:

$$FP(\%) = \frac{100}{rainfall\ amount} * ((S_{before} - S_{after}) - 1) * V_{0-2cm} \tag{1}$$

The rainfall amount was measured with the flow meter during the experiment. Sbefore and Safter represent the mean absolute salinity before and after the rainfall phase during the 15-minute periods, respectively. V_{0-2cm} is the volume of the water mass inside the tank of the first 2 cm.

Calculation of fractional change of salt content

We used the following equation to calculate the fractional change of salt content at the different depths after the rainfall of the experiment with a turbulent-free waterbody:

$$Fractionalchange = \frac{(S_R - S_{rain})}{(S_0 - S_{rain})} \tag{2}$$

The initial salinity is described with S_0 and the salinity after the artifial rain with S_R. The salinity of the artifical rain water (S_{rain}) is expexted to be 0. A result of 1 indicates no change in salt content.

Supporting figures and text

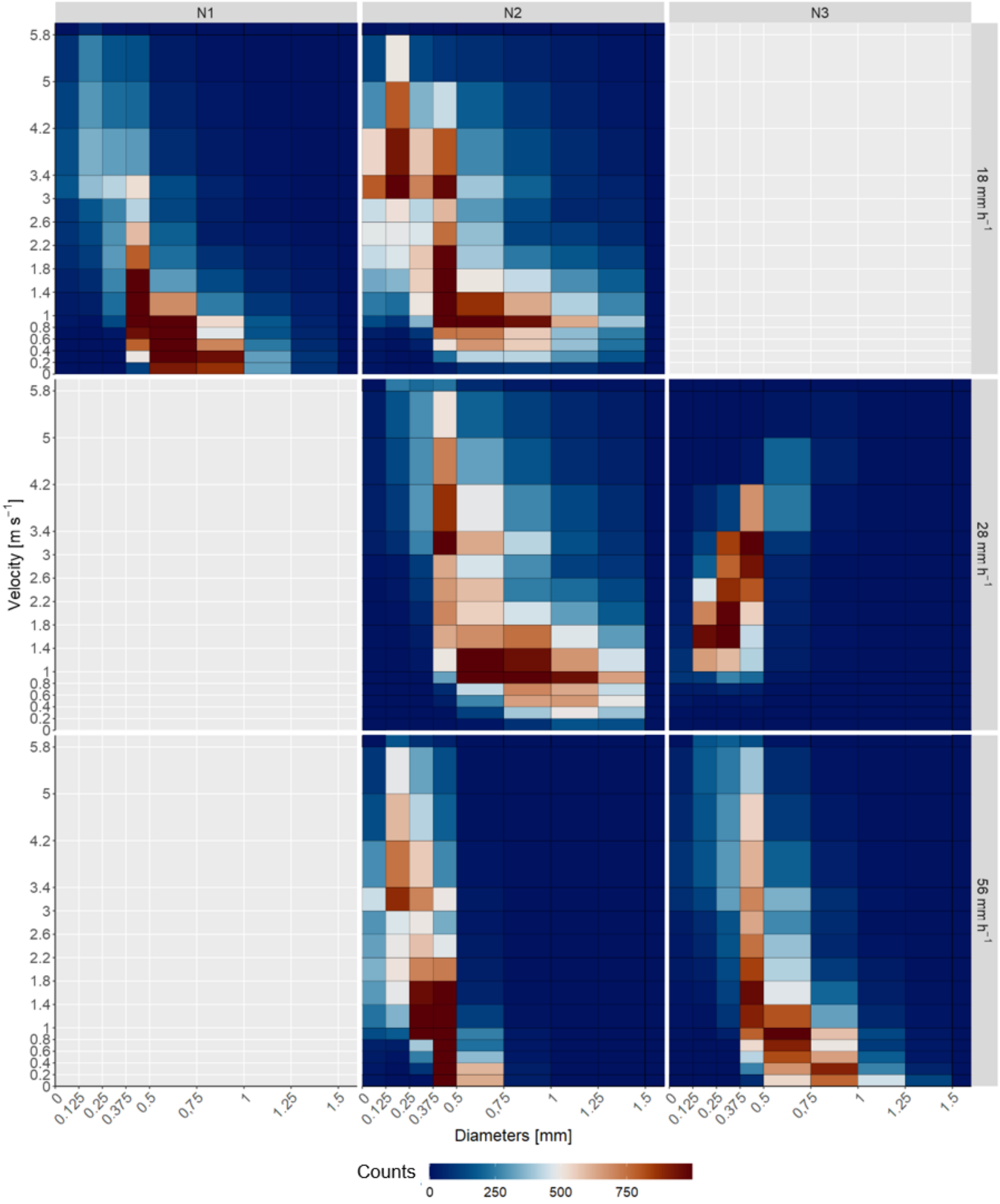

Figure S1: Distribution of droplet sizes and velocities of the three nozzle types N1, N2, and N3 and rain intensities 18 mm h^{-1}, 28 mm h^{-1}, and 56 mm h^{-1} directly under the nozzle. The colors show the mean number of droplets of the size and velocity categories of 1 minute.

During the phase of no mixing, stratification developed after rainfall at all three intensities of 18, 28, and 56 mm h^{-1}. The positive ΔT at the surface shows that the temperature of the artificial rain was higher than that of seawater. The anomalies of N2 with 28 mm h^{-1} are higher compared to the anomalies of N3 with 56 mm h^{-1}. The mean temperature of the rain was different with 17.54 °C, 17.44 °C, and 15.27 °C at the intensities of 18 mm h^{-1}, 28 mm h^{-1}, and 56 mm h^{-1}, respectively.

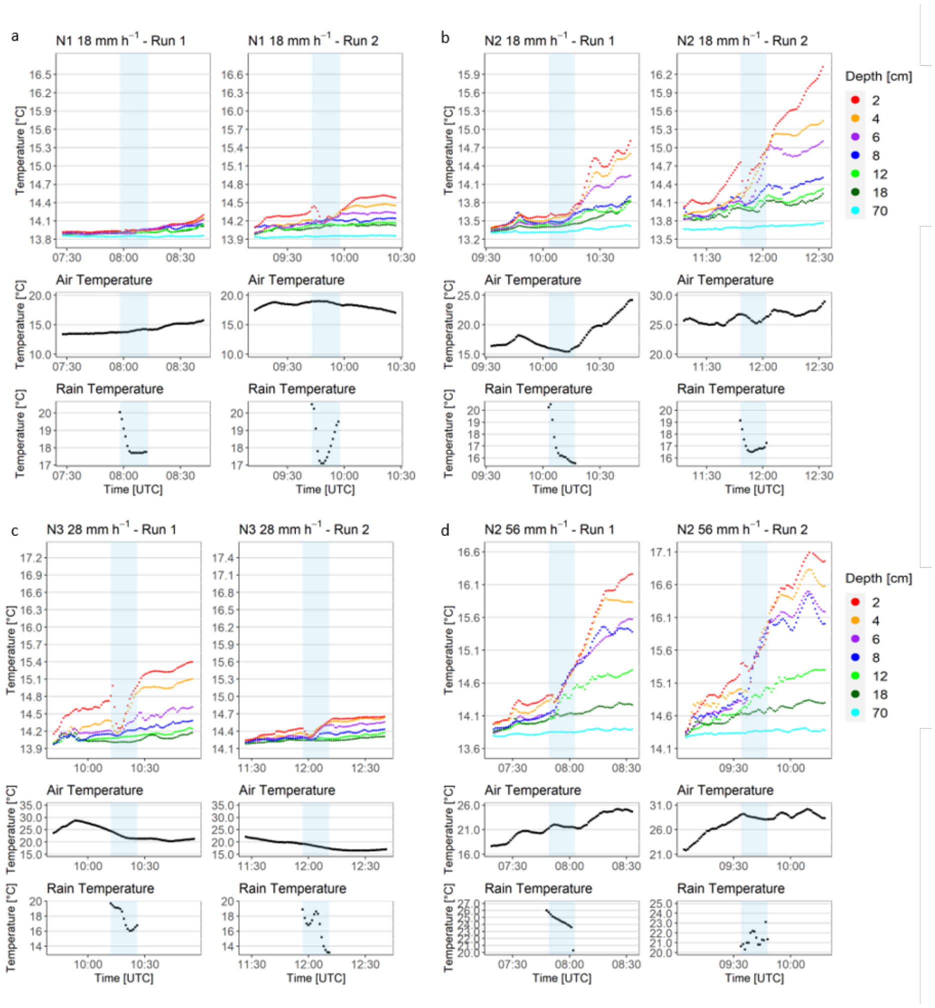

Figure S2: Time series of temperature changes during different rain scenarios with the first and second runs of experiment one (turbulence-free water body). The light blue rectangle indicates the 15-minute rainfall period.

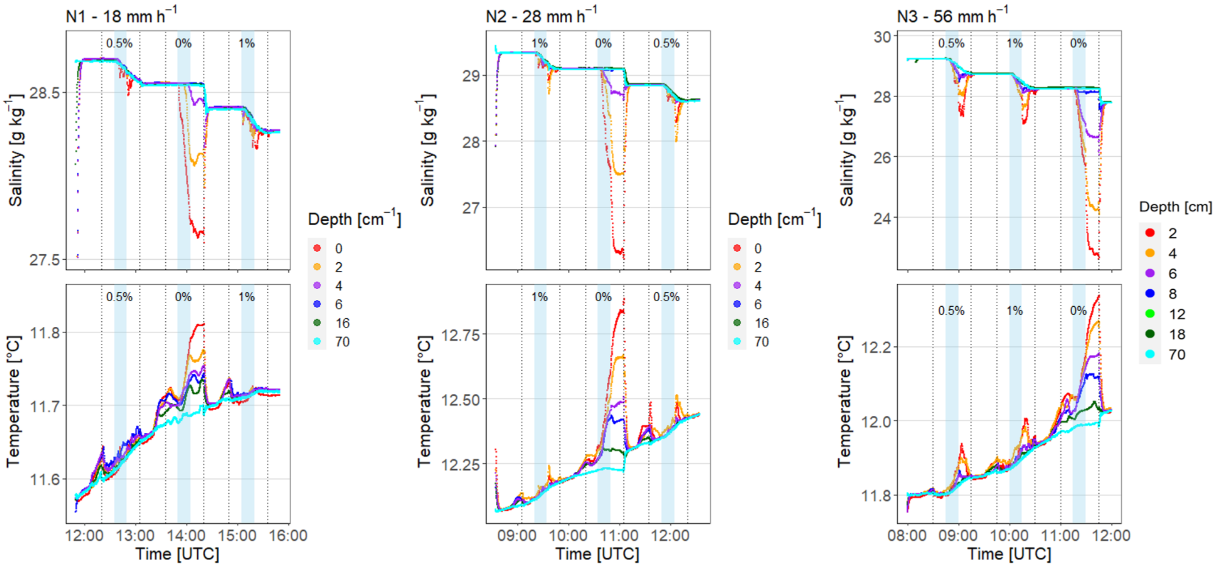

Figure S3: Time series of salinity and temperature changes during different rains in experiment two (turbulence-mixed water body). The light blue rectangle indicates the 15-minute rainfall period. The dotted lines indicate the start and stop of the 45 minutes with a certain pump level of 0 %, 0.5 %, and 1 %.

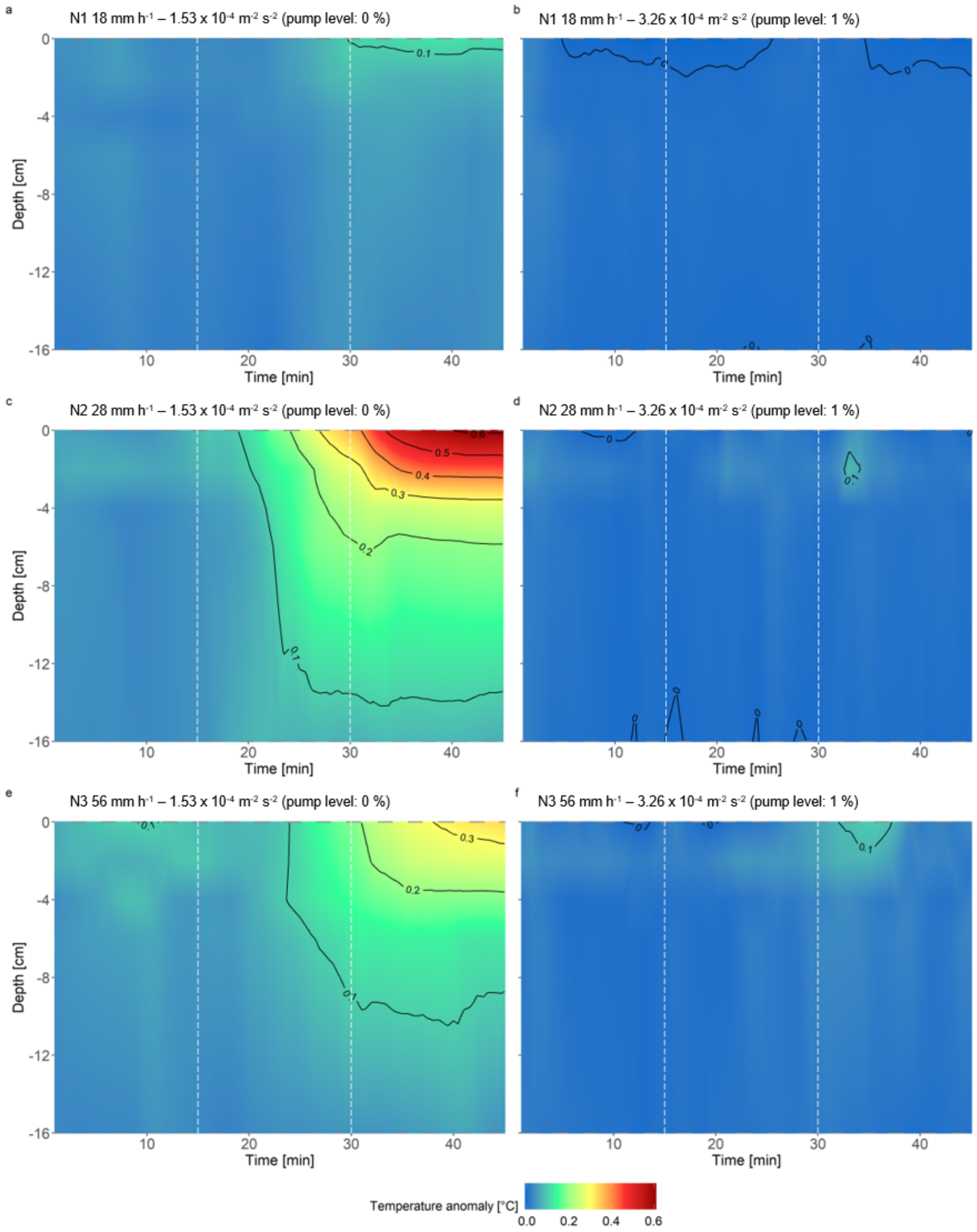

Figure S4: Contour plot of ΔT at different depths during three rainfall scenarios in the second experiment with a turbulence-free and mixed water body. Shown are the lowest intensity of 18 mm h^{-1} and N1 (a-b), the medium intensity of 28 mm h^{-1} and N2 (c-d), and the highest intensity of 56 mm h^{-1} and N3 (e-f) with a pump level of 0 % and 1 %. The start and end times of the precipitation phase are indicated with a dashed line (minutes 16–30). The black isolines show the progression of equal temperatures with time and depth.

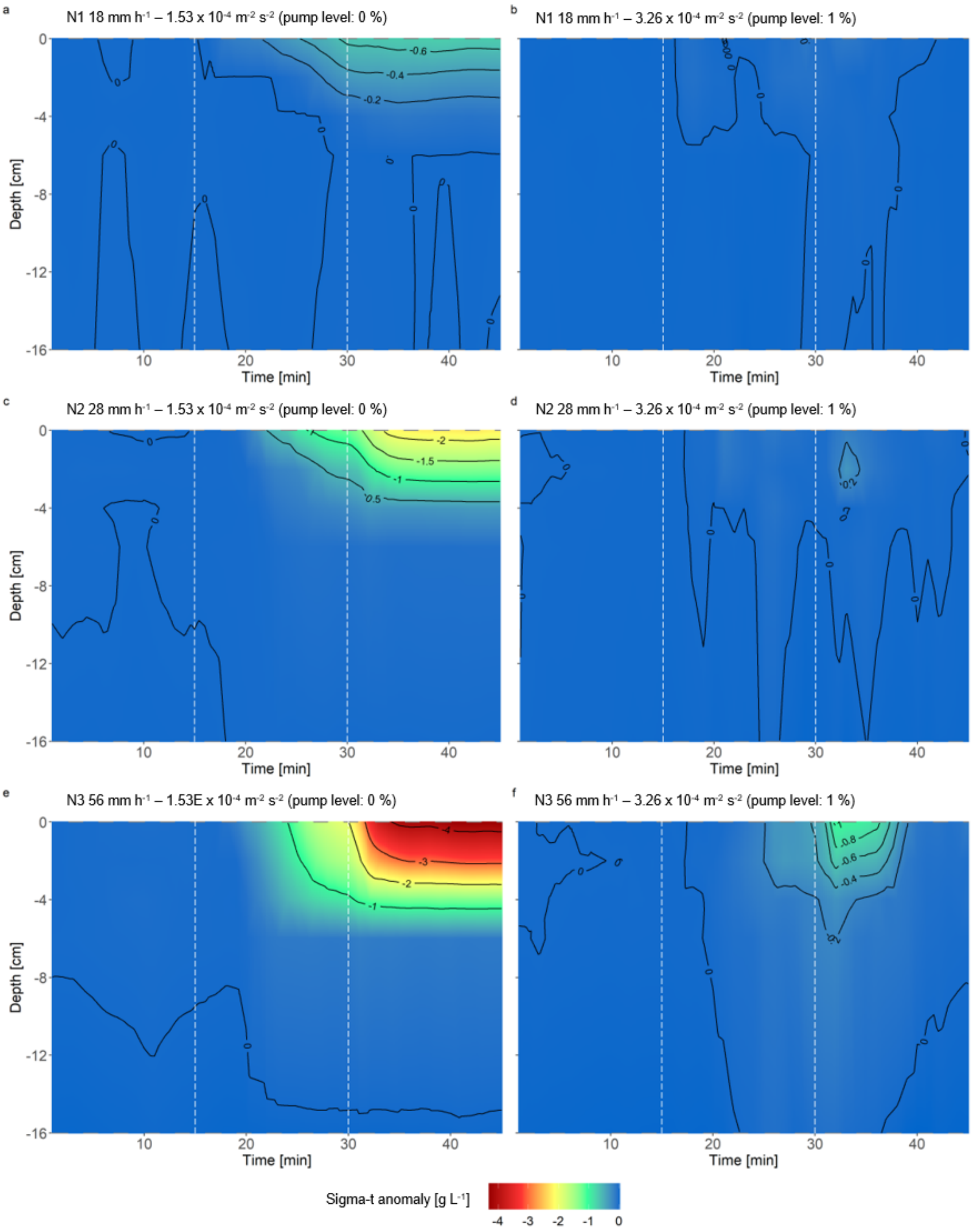

Figure S5: Contour plot of ΔD at different depths during three rainfall scenarios of the second experiment with a turbulence-mixed water body. Shown are the lowest intensity of 18 mm h^{-1} and N1 (a-b), the medium intensity of 28 mm h^{-1} and N2 (c-d), and the highest intensity of 56 mm h^{-1} and N3 (e-f) with pump levels of 0 %, 0.5 %, and 1 %. The start and end times of the precipitation phase are indicated with a white dashed line (minutes 16–30). The black isolines show the progression of equal sigma-t densities with time and depth.

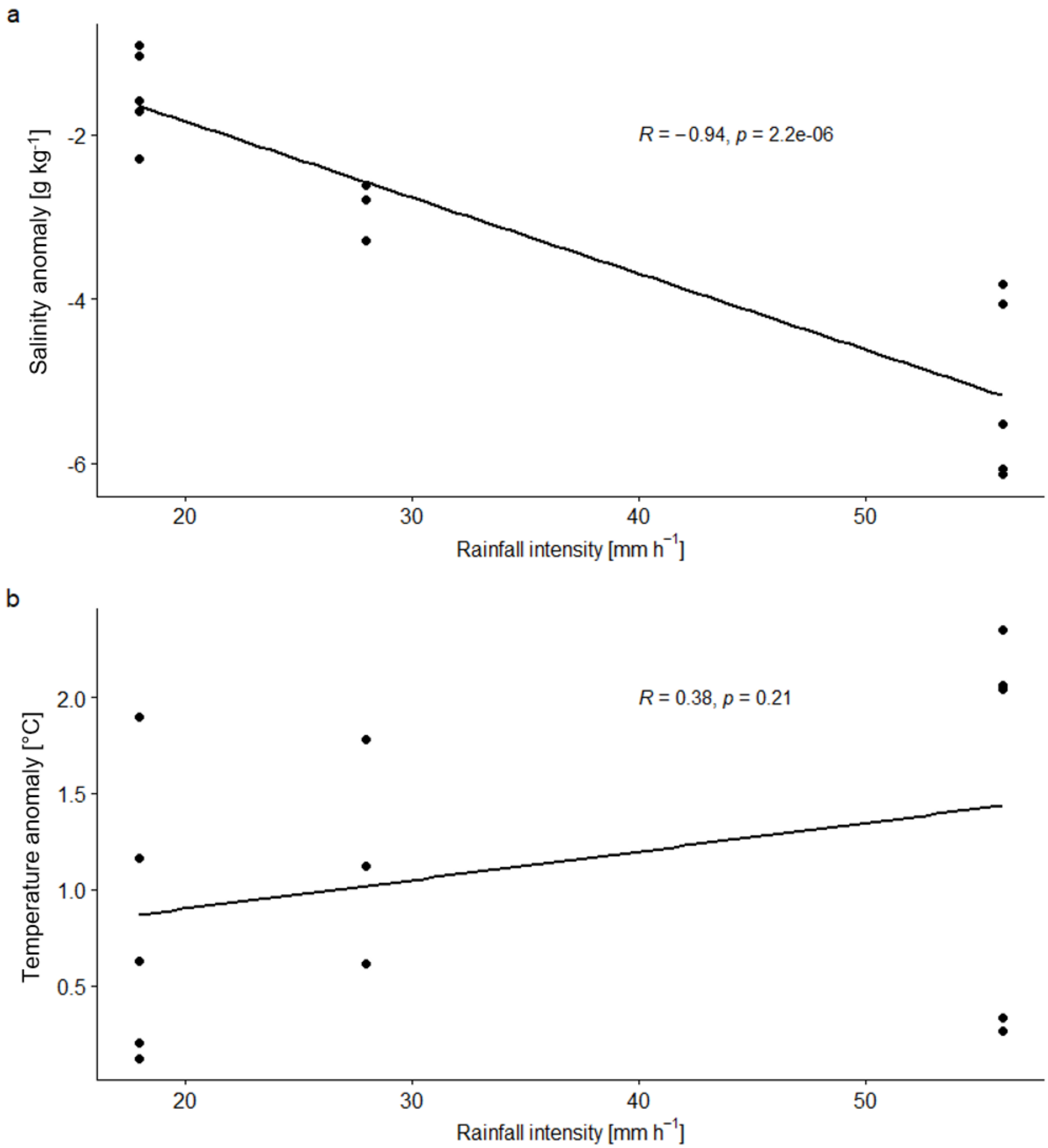

Figure S6: Scatter plot showing the correlation between the rainfall intensity and the maximum salinity (a) and temperature anomalies (b) at a depth between 0 and 2 cm during the first experiment and the turbulent-free parts of the second experiment. n = 13.

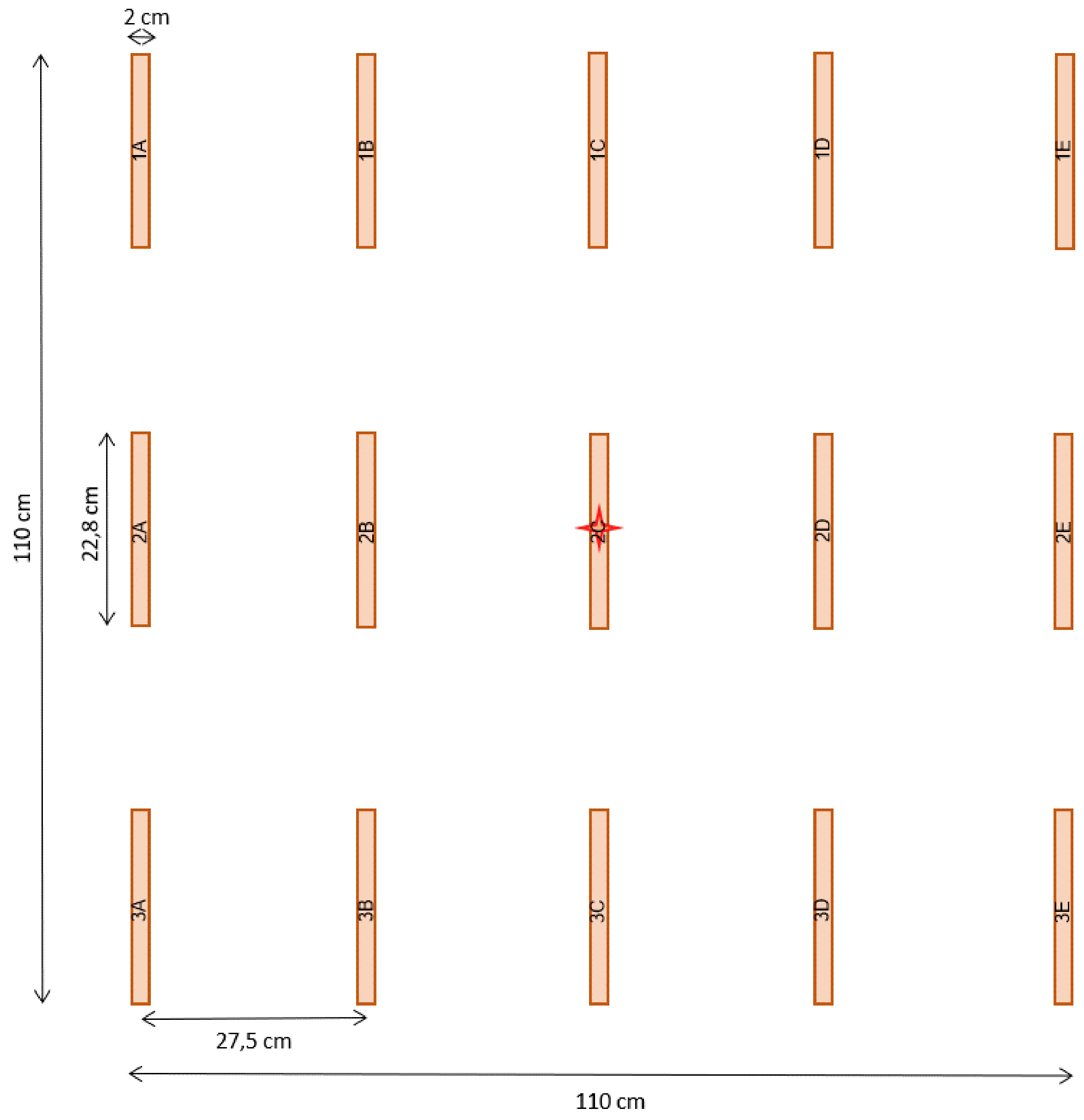

Figure S7: 15 Positions of the optical rain sensor 1A–3E. Each red rectangle represents the area of the laser generated by the distrometer to measure rainfall intensity and droplet properties, such as drop sizes and velocities. The red star indicates the position of the nozzle above the calibration area.

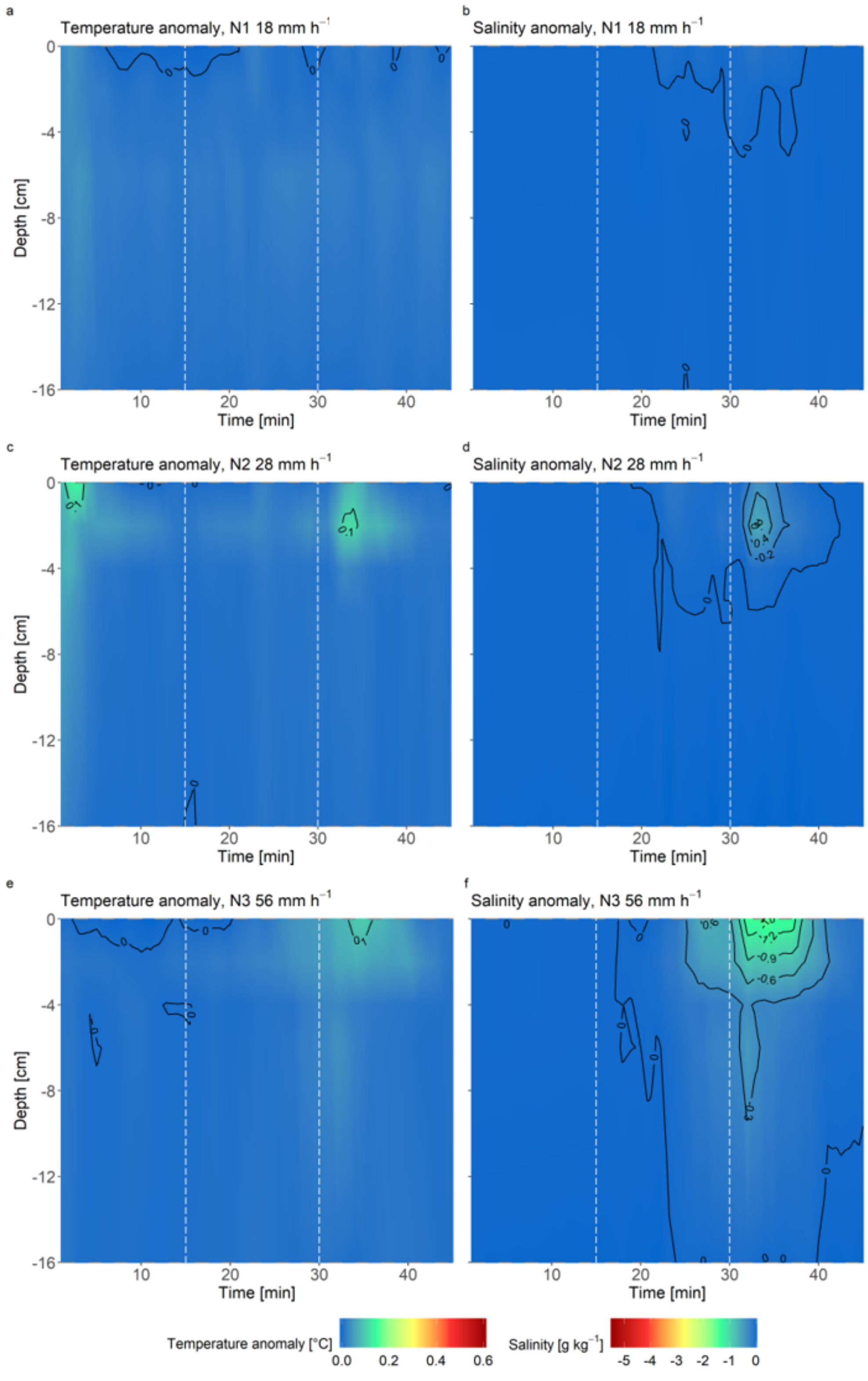

Figure S8: Contour plot of ΔS and ΔT at different depths of the second experiment with a turbulence-mixed water body. Shown are ΔS (a) and ΔT (b) of the lowest intensity of 18 mm h^{-1} and N1, the medium intensity of 28 mm h^{-1} and N2 with ΔS (c) and ΔT (d), and the highest intensity of 56 mm h^{-1} and N3 with ΔS (e) and ΔT (f) with a mean TKE of 3.37 x 10^{-4} m^{-2} s^{-2} (pump level: 0.5 %). The start and end times of the precipitation phase are indicated with a white dashed line (minutes 16–30). The black isolines show the progression of equal sigma-t densities with time and depth.

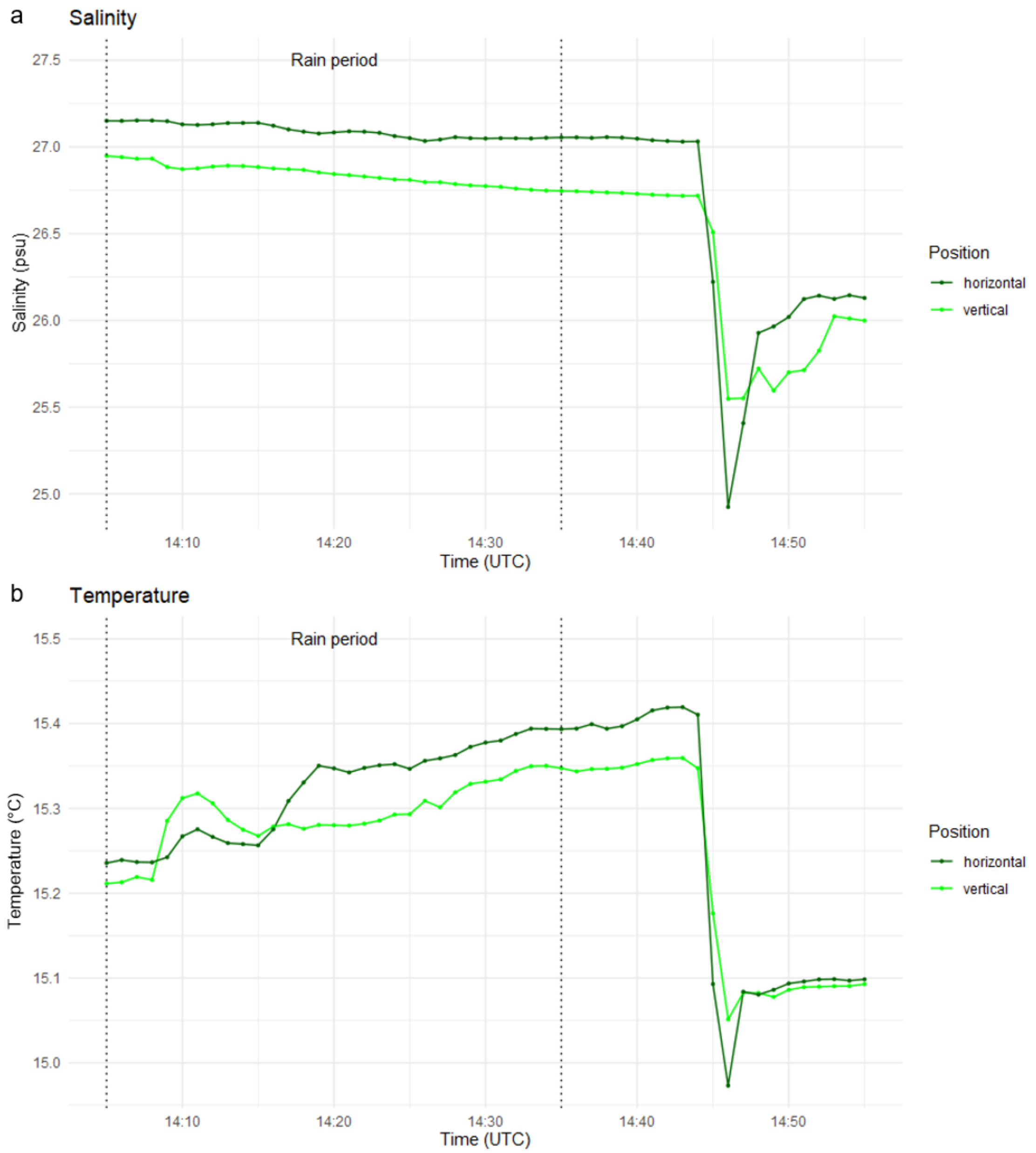

Figure S9: Timeseries of a test run with captured salinity and temperature by to different mounted CTDs at 8 cm depth with an rain intensity of 56 mm h^{-1} with N3. One is mounted horizontal and one vertical in the water of the mesocosm tank. Rain fall was applied for 30 minutes to the tank.
